# Psychometric analysis of new lymphoma-specific patient-reported symptom measures derived from the EORTC item library

**DOI:** 10.1186/s41687-026-01126-w

**Published:** 2026-06-22

**Authors:** Angély Loubert, Kristin Creel, Naleen Raj Bhandari, Lisa M. Hess, Amy S. Ruppert, Paolo Abada, Antoine Regnault, Nalin Payakachat

**Affiliations:** 1 Modus Outcomes, A Division of THREAD, Cambridge, MA USA; 2 Eli Lilly and Company, Indianapolis, IN USA

## Abstract

**Introduction:**

Three patient-reported outcome (PRO) measures specific to lymphoma symptoms were previously defined using mixed-methods research, comprising questions from the European Organization for Research and Treatment of Cancer (EORTC) Quality of Life Questionnaire Core 30 items (QLQ-C30) and Item Library (IL): measures of Chronic Lymphocytic Leukemia/Small Lymphocytic Lymphoma (CLL/SLL) Symptoms, Mantle Cell Lymphoma (MCL) Symptoms, and an Expanded Fatigue measure. The objective of this study was to assess psychometric properties of the measures and provide guidance for interpretation by identifying thresholds for determining meaningful within-patient change (MWPC).

**Methods:**

Psychometric properties of the CLL/SLL-related Symptoms, MCL-related Symptoms, and Expanded Fatigue measures were assessed using classical test theory (CTT) and Rasch measurement theory (RMT) using blinded data collected from two phase 3 trials in participants with CLL/SLL (BRUIN-CLL-321, *N* = 211) and MCL (BRUIN-MCL-321, *N* = 523). CTT analyses assessed reliability, construct validity and ability to detect change, while RMT analyses explored adequacy of summing items to generate summary scores. MWPC for each score was estimated using anchor-based and distribution-based methods.

**Results:**

The three measures demonstrated adequate internal consistency and test-retest reliability, and strong evidence of construct validity. Each showed a good ability to detect worsening and, to a lesser degree, improvement. RMT analyses confirmed adequacy of the response scales but suggested suboptimal targeting for participants with the lowest symptoms severity. For the two symptoms-related measures, items related to fatigue were over-discriminating, suggesting that they are driving the measures. Analyses of MWPC resulted in the following estimates: for the CLL/SLL-related Symptoms score, an improvement of -15.38 points (worsening unable to be estimated); for the MCL-related Symptoms score, an improvement of -10.25 points and a worsening of 5.12 points; and for the Expanded Fatigue score, an improvement -16.66 points in both CLL/SLL and MCL populations and a worsening of 11.11 points in participants with MCL (unable to be estimated in CLL/SLL).

**Conclusion:**

The CLL/SLL-related Symptoms, MCL-related Symptoms, and Expanded Fatigue measures showed strong psychometric properties in both CTT and RMT frameworks. Combined with previously published qualitative data, this research showed that the measures are appropriate for use in clinical trials of participants with CLL/SLL and MCL.

**Trial registrations:**

BRUIN-CLL-321, NCT04666038. Registered 07 December 2020, https://www.clinicaltrials.gov/study/NCT04666038; BRUIN-MCL-321, NCT04662255, Registered 04 December 2020, https://clinicaltrials.gov/study/NCT04662255.

**Supplementary Information:**

The online version contains supplementary material available at 10.1186/s41687-026-01126-w.

## Introduction

Patient-reported outcomes (PROs) are vital to ongoing efforts to improve care and outcomes for cancer patients and their use in clinical trials helps patients and providers understand added benefits and risks of new treatments. As disease-related symptoms are experienced by patients, they should be captured by PROs in oncology clinical trials [1]. It is, therefore, essential to use PROs that target the specific symptoms experienced by the patient population.

Patients with B-cell malignancies such as chronic lymphocytic leukemia/small lymphocytic lymphoma (CLL/SLL; CLL from hereon) and mantle cell lymphoma (MCL) present with an array of diverse symptoms. In CLL, patients report experiencing fatigue, weight loss, lymphadenopathy, night sweats, fever, malaise, bleeding, and recurrent or persistent infections [2–4]. In MCL, symptoms include abdominal discomfort, fever, night sweats, and weight loss [5]. Fatigue related to cancer treatment is another common concern in CLL [6, 7] and MCL [8–10]. Patients with CLL or MCL typically experience recurrent disease trajectories and multiple cycles of treatments with or without long “treatment holidays”. These symptoms can thus have a profound impact on patients and their daily lives.

There are few PRO measures that specifically assess symptoms of B-cell malignancies. Measures, such as, European Organization for Research and Treatment of Cancer’s (EORTC) Quality of Life Questionnaires (QLQ-CLL17 in patients with CLL [11], QLQ-NHL-HG29 in high-grade non-Hodgkin lymphoma (NHL) [12], and QLQ-NHL-LG20 in low-grade NHL [12]), focus on broad concepts such as health-related quality of life and do not reflect key disease symptoms that are relevant to patients [4, 11]. Additionally, these existing measures have not been fully validated in a relapsed/refractory population, such as that in the current trials, or not specifically designed for patients with CLL or MCL. Consequently, mixed-methods research was conducted, including patient and clinician interviews and quantitative analyses [4], to inform the current study. The prior mixed-methods research led to the creation of conceptual models describing symptoms that are relevant for patients with CLL or MCL and established item sets that can be used to assess patient-reported symptom endpoints in clinical trials of CLL and MCL. Three PRO measures were derived comprising questions from the EORTC QLQ Core 30 items (QLQ-C30) and from the EORTC Item Library (IL): a CLL/SLL-related Symptoms measure, an MCL-related Symptoms measure, and an Expanded Fatigue measure.

The objective of this study was to demonstrate the psychometric properties of these three new measures in the context of clinical trials in patients with relapsed/refractory CLL and MCL and to identify thresholds of meaningful within-patient change (MWPC).

## Methods

### Data

This study was conducted using blinded data collected in two clinical trials studying pirtobrutinib compared to global standards of care in CLL (BRUIN-CLL-321) and MCL (BRUIN-MCL-321). BRUIN-CLL-321 is a phase 3 global, randomized, open-label study comparing pirtobrutinib as continuous monotherapy (Arm A) to Investigator’s choice of either continuous Idelalisib plus eight infusions of rituximab (IdelaR) or six cycles of Bendamustine plus rituximab (BR) (Arm B) in patients with CLL who had been treated with a covalent Bruton tyrosine kinase (BTK) inhibitor [13]. BRUIN-MCL-321 is a phase 3 global, randomized, open-label study comparing pirtobrutinib as continuous monotherapy (Arm A) with Investigator’s choice of an approved cBTKi (ibrutinib, acalabrutinib, and zanubrutinib) as continuous monotherapy (Arm B) in previously treated, BTKi-naïve patients with MCL [14].

### Patient-reported outcome (PRO) measures

In both studies, PRO endpoints were assessed using measures derived from the EORTC QLQ-C30 and from the EORTC IL. The EORTC QLQ-C30 is a 30-item scale used to assess quality of life (QoL), general functioning, and symptoms in cancer patients [15]. The QLQ-C30 includes five functional scales (physical, role, cognitive, emotional and social) and three symptom scales (fatigue, pain and nausea/vomiting) as well as six individual symptom items (dyspnea, insomnia, appetite loss, constipation, diarrhea, and financial difficulties). Additionally, the EORTC provides an IL, which is a database that compiles over 1,000 individual items that were created as part of the multiple development of cancer-specific modules within the EORTC family of PRO measures. Relevant items from the QLQ-C30 and the IL were used to derive two cancer-related symptom measures specific to CLL and MCL and a measure for fatigue which expanded the QLQ-C30 fatigue scale (from three to six items). Item composition of these measures was supported by a conceptual model of disease-related symptoms derived from a mixed-method analysis including literature review, clinician consultations, and qualitative interviews with patients with CLL and MCL [4]. Fatigue and other symptoms were highlighted as those impacting physical, role, and other functioning in this mixed-method analysis [4]. These three measures, along with the QLQ-C30 physical functioning (PF) scale, were used to assess PRO endpoints in the BRUIN-CLL-321 and BRUIN-MCL-321 trials. The composition of the derived scales is provided in Table 1.

**Table 1:** Composition of PRO measures derived from the EORTC QLQ-C30 and Item Library

	CLL/SLL-related Symptoms (IL87)	MCL-related Symptoms (IL88)	Expanded Fatigue
EORTC QLQ-C30	Were you short of breath?	Were you short of breath?	-
	Did you need to rest?	Did you need to rest?	Did you need to rest?
	Have you felt weak?	Have you felt weak?	Have you felt weak?
	Were you tired?	Were you tired?	Were you tired?
	Have you had pain?	Have you had pain?	*-*
	Have you had trouble sleeping?	-	*-*
	Have you lacked appetite?	Have you lacked appetite?	*-*
	Have you felt nauseated?	Have you felt nauseated?	*-*
EORTC Item Library	Have you had a lack of energy?	Have you had a lack of energy?	Have you had a lack of energy?
	Have you felt drowsy?	Have you felt drowsy?	Have you felt drowsy?
	Have you had sudden tiredness?	Have you had sudden tiredness?	Have you had sudden tiredness?
	Have you had fevers or chills?	Have you had fevers or chills?	-
	Did you have night sweats?	Did you have night sweats?	-
	-	Have you had a bloated feeling in your abdomen?	-

The measure of CLL/SLL-related Symptoms was derived from 13 items including *dyspnea*, *fatigue* (three items), *general pain*, *insomnia*, *lack of appetite*, and *nausea* from the QLQ-C30, along with five items (IL58: *night sweats*, *fever/chills*, and *fatigue* (three items) from the IL. This 13-item set capturing CLL/SLL-related symptoms is labeled as IL87 in the EORTC IL system. The measure of MCL-related Symptoms was also derived from 13 items including *dyspnea*, *fatigue* (three items), *general pain*, *lack of appetit*e, and *nausea* from the QLQ-C30, along with six items (IL63: *night sweats*, *fever/chills*, *fatigue* (three items) and *bloating of the abdomen* from the IL. This 13-item set capturing MCL-related symptoms is labeled as IL88 in the EORTC IL system. Alternative versions of both disease-related measures were also explored, including the exclusion of items related to B symptoms (*lack of appetite*, *fever*, *night sweats*). Three items (*need to rest*, *felt weak*, *were tired*) from the QLQ-C30 Fatigue measure and three additional items (*lack of energy*, *felt drowsy*, *sudden tiredness*) from the IL were used to derive a measure specific to the experience of fatigue (referred to as Expanded Fatigue).

The three new PRO measures were scored according to the same principles described in the scoring manual for the QLQ-C30 [16], with a range of 0 to 100. Higher scores reflect a higher level of symptomatology for each of the PRO measures in this study.

The items from the three new measures were collected at baseline (week 1) and at the end of each four-week cycle (either as part of the QLQ-C30 and IL58/IL63, collected every 12 weeks, or as part of the IL87/IL88, collected at cycles where the full QLQ-C30 was not collected). The EORTC IL for Physical Functioning (IL19), a Patient Global Impression of Severity (PGI-S)—Cancer Symptoms, and a Patient Global Impression of Change (PGI-C)—Cancer Symptoms were administered at baseline (week 1, with the exception of the PGI-C), and then at the beginning of each treatment cycle (every 4 weeks), until the end of treatment (EOT) in both clinical trials.

### Psychometric analysis

Psychometric properties of the two derived measures for disease-related symptoms, CLL/SLL-related Symptoms and MCL-related Symptoms, and the measure of Expanded Fatigue (as described in Table 1), were assessed using classical test theory (CTT) and Rasch Measurement Theory (RMT) methods. A summary of the analyses that were conducted is provided in Table 2.

CTT analyses assessed reliability, construct validity, and ability to detect change in scores of the three PRO measures. Assessment of reliability included internal consistency using Cronbach’s alpha and test-retest reliability using intraclass correlation coefficients (ICCs) between baseline and week 5 for participants with CLL and between baseline and week 9 for participants with MCL (‘Cycle 2’ assessment, from both trials). Reliability coefficients were interpreted as follows: unsatisfactory reliability (< 0.70), modest reliability (0.70–0.79), adequate reliability (0.80–0.89), good reliability (0.90–0.95), and excellent reliability (> 0.95) [17].

Construct validity was evaluated by estimating the item-to-item and item-to-scale (total measure score) correlations for each PRO score and examining the correlations between the score and other variables, including PGI-S, Eastern Cooperative Oncology Group (ECOG) Performance Status, laboratory parameters, and four scales of the EQ-5D-5 L (mobility, self-care, usual activities, and the visual analogue scale [VAS]) at week 1 (baseline). Each of the PRO scores were hypothesized to have a moderate to high association (*r* > 0.40) [18, 19] with the PGI-S, a low to moderate association (0.20 ≤ *r* ≤ 0.69) with the ECOG Performance Status (evaluated for participants with MCL, only), a low association (0.20 ≤ *r* ≤ 0.39) with laboratory parameters, and a low to moderate association (0.20 ≤ *r* ≤ 0.69) with the EQ-5D-5 L dimensions.

The ability of the PRO measures to detect change over time was evaluated between week 1 and week 9 (the timepoint which included the highest number of participants with improved and worsened categories according to the change in PGI-S score), by computing the effect size (ES) observed in subgroups of participants categorized as “improved”, “stable”, and “worsened” according to their change in PGI-S response and to their PGI-C response, separately. ES were interpreted according to Cohen’s recommendations: negligible (<|0.20|), small (|0.20|–|0.49|), medium (|0.50|–|0.79|), and large (≥|0.80|) [20].

RMT analyses explored whether the items from each of the three measures were targeted to the population of the BRUIN-CLL-321 and BRUIN-MCL-321 studies and the legitimacy of summing items to generate a summary score that quantifies the concept of interest (disease-related symptoms) [21]. The RMT approach [21–23] examines the extent to which the observed data (actual responses to items) fit predictions of the responses expected from the Rasch model (that defines how a set of items should perform to generate reliable and valid measurements [22]). For these analyses, data from all available assessment timepoints were pooled (stacked data), as allowed by the parameter separation property of the Rasch model [24]. The following RMT-based psychometric properties were examined: suitability of the response scale (functioning of response options) [25], item performance [23], local independence of items [23], person fit [23], targeting [26, 27], and reliability (evaluated by the person separation index [PSI] [28]; see guidance for interpretation of reliability coefficients above).

To aid in the interpretation of the PRO scores, analyses were conducted to confirm thresholds for MWPC for each measure (CLL/SLL-related Symptoms, MCL-related Symptoms, and Expanded Fatigue). MWPC thresholds for improvement based on these scores were previously determined in analyses based on data from LOXO-BTK-18001 study, which was a phase 1/2 study of pirtobrutinib in participants with CLL and MCL. A negative change of at least 15.38 in the CLL/SLL-related Symptoms score, a negative change of at least 10.25 in the MCL-related Symptoms score, and a negative change of at least 16.66 in the Expanded Fatigue score were found to be MWPC thresholds for improvement in that analysis [29, 30]. The appropriateness of these thresholds was thus confirmed based on data from BRUIN-CLL-321 and BRUIN-MCL-321 using anchor-based methods (considering PGI-S and PGI-C as potential anchors) and supportive distribution-based methods. Analyses were performed at the post-baseline visits that maximized the number of participants that improved and worsened according to the PGI-S (i.e., week 9 for both studies).

**Table 2:** Summary of analyses conducted for the CLL/SLL-related Symptoms, MCL-related Symptoms, and Expanded Fatigue scores using data from BRUIN-CLL-321 and BRUIN-MCL-321

CTT Analyses	RMT Analyses	MWPC Analyses
• Internal consistency reliability • Test-retest reliability • Construct validity • Ability to detect change	• Suitability of the response scale • Item performance • Local independence of items • Person fit • Targeting • Reliability	• Anchor-based methods • Distribution-based methods

CTT=classical test theory; RMT=Rasch measurement theory; MWPC=meaningful within-patient change

## Results

The sample from BRUIN-CLL-321 included 211 participants with CLL (data cutoff of 13 July, 2023) and the sample from BRUIN-MCL-321 included 523 participants with MCL (data cutoff of 29 March, 2024).

### Psychometric evidence: classical test theory

Full results from the CTT analyses are summarized in Table 3. Assessment of internal consistency reliability of the three measures assessed was adequate to good [17]. Cronbach’s alpha at baseline was 0.91 for the CLL/SLL-related Symptoms measure, 0.89 for the MCL-related Symptoms measure, and 0.93 for the Expanded Fatigue measure among participants with CLL and 0.90 among participants with MCL. Test-retest reliability was modest to adequate [17] in participants indicating being stable on the PGI-S (i.e., no change in PGI-S rating between assessments), with ICCs of 0.78 for CLL/SLL-related Symptoms (between baseline and week 5), 0.73 for MCL-related Symptoms (between baseline and week 9), and 0.80 for Expanded Fatigue among participants with CLL (between baseline and week 5) and 0.79 among participants with MCL (between baseline and week 9).

Assessment of construct validity of the CLL/SLL-related Symptoms and MCL-related Symptoms measures showed that most item-to-item correlations ranged from low to high (*r* ≤ 0.88). The item pairs with the highest correlations were those related to fatigue and those with the lowest correlations were related to nausea, lack of appetite, night sweats, and fever or chills. In the Expanded Fatigue measure, item-to-item correlations were all moderate to high (*r* > 0.70 between most items) in both populations. Item-to-scale correlations ranged from *r =* 0.34 to *r =* 0.83 in the CLL/SLL-related Symptoms measure and from *r* = 0.33 to *r* = 0.79 in the MCL-related Symptoms measure. For both symptoms scores, items related to fatigue showed the highest correlations with the overall score while those related to nausea and fever or chills had the lowest correlations. Item-to-score correlations for the Expanded Fatigue measure ranged from *r* = 0.63 to *r* = 0.82 among participants with CLL and *r* = 0.58 to *r* = 0.82 among participants with MCL. Detailed information about correlations can be found in the Supplementary materials, Suppl. Table 1 for CLL/SLL-related Symptoms, Suppl. Table 2 for Expanded Fatigue for participants with CLL, Suppl. Table 3 for MCL-related Symptoms, and Suppl. Table 4 for Expanded Fatigue for participants with MCL.

Association of the three measures with other variables were generally as hypothesized. For the CLL/SLL-related Symptoms measure, the correlation coefficient with the PGI-S was high (*r* = 0.70), with the ECOG Performance Status was low (*r* = 0.38), and with laboratory values were low or very low (hematocrit: *r*=-0.17, platelet count: *r*=-0.06, and LDH: *r* = 0.19). For the MCL-related Symptoms measure, the correlation coefficient with the PGI-S was moderate (*r* = 0.57), with the ECOG Performance Status was very low (*r* = 0.18), and with laboratory values were low or very low (hematocrit: *r*=-0.26, platelet count: *r*=-0.07, and LDH: *r* = 0.22). The MCL-related Symptoms measure also had moderate correlations with the EQ-5D-5 L Mobility (*r* = 0.49), Self-care (*r* = 0.35), and Usual Activities (*r* = 0.61) dimensions, as well as with the VAS score (*r*=-0.59) (correlations between the CLL/SLL-related Symptoms score and EQ-5D-5 L dimensions were not calculated). For the Expanded Fatigue measure, the correlation coefficients with the PGI-S were moderate (*r* = 0.69 among CLL participants and *r* = 0.53 among MCL participants), with the ECOG Performance Status was low (*r* = 0.36 among participants with CLL) and very low (*r* = 0.17 among participants with MCL), and with laboratory values were low or very low (hematocrit: *r*=-0.17, platelet count: *r*=-0.07, and LDH: *r* = 0.20 among participants with CLL, and *r*=-0.23, *r*=-0.07, and *r* = 0.19, respectively, among participants with MCL). The Expanded Fatigue measure among participants with MCL had low to moderate correlations with the EQ-5D-5 L dimensions: Mobility (*r* = 0.48), Self-care (*r* = 0.33), Usual Activities (*r* = 0.60), VAS score (*r*=-0.57).

The measures’ ability to detect change was assessed using the change in PGI-S and the PGI-C as anchors. For the CLL/SLL-related Symptoms measure assessed between baseline and week 9, results showed a medium ES of -0.73 for participants who improved when using the PGI-S but a small ES of -0.38 when using the PGI-C. Results showed a negligible ES of 0.14 for participants that worsened based on PGI-S while too few participants worsened according to the PGI-C to interpret the results. For the MCL-related Symptoms measure assessed between baseline and week 9, a medium ES of -0.52 was found for participants who improved on the PGI-S, but a small ES of -0.20 was found for those who improved on the PGI-C. For the MCL-related Symptoms measure assessed between baseline and week 9, results showed a large ES of 0.86 for participants whose PGI-S worsened while too few participants worsened according to the PGI-C to interpret the results. For both scores, negligible ES were obtained for participants categorized as stable according to both the PGI-S and the PGI-C (CLL/SLL-related Symptoms: -0.08 and -0.10; MCL-related Symptoms; -0.14 and 0.12). For the Expanded Fatigue measure, using the PGI-S as an anchor, the ES was medium for participants with CLL (-0.62) and small for those with MCL (-0.36) who were categorized as improved. Using the PGI-C as an anchor, ES was -0.28 among participants with CLL and -0.10 among participants with MCL who improved. For participants who were categorized as worsened according to the PGI-S, the ES was negligible (0.18) for CLL and medium (0.69) for MCL (too few participants worsened according to the PGI-C to interpret the results).

**Table 3:** Summary of CTT results

Score	Reliability		Construct Validity							Ability to Detect Change		
	Cronbach’s alpha	ICC^1^	Item-to-item correlations (range)	Item-to-scale correlations (range)	Correlation with other variables^2^					Standardized Effect Size		
					PGI-S	ECOG	Hematocrit	Platelet count	LDH	Worsened^3^	Stable^3^	Improved^3^
CLL/SLL-related Symptoms	0.91	0.78	0.21–0.88	0.34–0.83	0.70	0.38	-0.17	-0.06	0.19	0.14	-0.08	-0.73
MCL-related Symptoms	0.89	0.73	0.25–0.86	0.33–0.79	0.57	0.18	-0.26	-0.07	0.22	0.86	-0.14	-0.52
Expanded Fatigue (CLL)	0.93	0.80	0.62–0.88	0.63–0.82	0.69	0.36	-0.17	-0.07	0.20	0.18	0.00	-0.62
Expanded Fatigue (MCL)	0.90	0.79	0.57–0.86	0.58–0.82	0.53	0.17	-0.23	-0.07	0.19	0.69	-0.08	-0.36

^1^ ICC among stable participants, according to the PGI-S

^2^ Spearman correlation

^3^ Worsened, stable and improved according to the PGI-S

### Psychometric evidence: rasch measurement theory

**Fig. 1:**
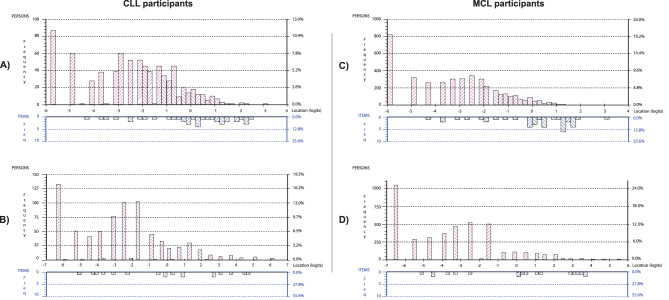
Targeting between the items and the sample for (**A**) the CLL/SLL-related Symptoms measure, (**B**) the Expanded Fatigue measure in CLL participants, (**C**) the MCL-related Symptoms measure, (**D**) the Expanded Fatigue measure in MCL participants. The upper panel (pink boxes) shows the distribution of the participant assessments over the continuum of symptoms/fatigue; the lower panel (blue boxes) shows the distribution of the items on the continuum of symptoms/fatigue

Assessing the targeting of the measures’ items to the study sample by comparing the distribution of the participants and the items over the symptom continuum revealed floor effects for each item. No item captured the severity of disease-related symptoms or fatigue experienced by participants with the lowest severity level. At the same time, few participants in the study had a level of severity that was measured by items that represented higher disease-related symptom severity. As shown in Fig. 1, where the distribution of participants is represented by pink bars and the distribution of items is represented by blue bars, 33% of participants had the lowest possible score for CLL/SLL-related Symptoms, 28% for MCL-related Symptoms, and 29% and 32% of participants with CLL and MCL, respectively, had the lowest possible score for Expanded Fatigue.

All response options for all the three measures (CLL/SLL-related Symptoms, MCL-related Symptoms, and Expanded Fatigue) had ordered thresholds, indicating that the response options worked as intended. As the severity of symptoms increased, the probability of choosing a higher (more severe) response option also increased (see Suppl. Figure 1). Based on the item hierarchy for the CLL/SLL-related and MCL-related Symptoms measures, items that were related to fatigue were experienced by participants who also recorded lower overall severity of symptoms (see Suppl. Figure 1). Items related to B symptoms (*fever or chills*,* night sweats*,* lack of appetite*) and *nausea* were generally endorsed by participants who scored higher on the measure.

In analyses of fit residuals, five items of the CLL/SLL-related Symptoms measure had fit residuals that were significantly outside of the recommended range (-2.5 to 2.5, all *p* < 0.0008, see Suppl. Table 8). Two items (*had trouble sleeping* and *had night sweats*) were under-discriminating i.e., they were not as sensitive as expected regarding differences in symptom severity, whereas three items (*felt weak*,* felt tired*, and *lack of energy*) were over-discriminating i.e., that they were overly sensitive to changes in symptoms. For the MCL-related Symptoms measure, 10 items had fit residuals that were significantly outside the recommended range (see Suppl. Table 9), including five that were under-discriminating (*had pain*,* lacked appetite*,* night sweats*,* fevers or chills*, and *bloated feeling in abdomen*) and five that were over-discriminating (*need to rest*,* felt weak*,* felt tired*,* lack of energy*, and *sudden tiredness*). In the Expanded Fatigue measure (see Suppl. Tables 10 and 11), all items had fit residuals that were within the recommended range (-2.5 to 2.5) among participants with CLL, while three items had fit residuals significantly outside the recommended range among participants with MCL (*felt tired*,* lack of energy*, and *felt drowsy*).

Reliability, as measured by the PSI, was adequate for each of the measures: PSI = 0.86 for CLL/SLL-related Symptoms, PSI = 0.81 for MCL-related Symptoms, PSI = 0.87 for Expanded Fatigue among participants with CLL, and PSI = 0.83 for Expanded Fatigue among participants with MCL.

### Evidence to support interpretation of the PRO scores

#### Evaluation of the association of the PRO scores with the selected anchor variables

In participants with CLL, Spearman rank correlation coefficients between the change in PGI-S and the change in the CLL/SLL--related Symptoms score and Expanded Fatigue score were *r =* 0.38 and 0.35, respectively. In participants with MCL, Spearman rank correlation coefficients between the change in PGI-S and the change in the MCL-related Symptoms score and Expanded Fatigue score were *r =* 0.43 and 0.37, respectively. These correlations were adequate (*r* > 0.3) [31] and supported the use of PGI-S as an anchor. Correlations of the change in these three PRO measures with the PGI-C were consistently below the 0.3 threshold, which is why PGI-C was not considered an acceptable anchor variable for the anchor-based methods.

#### Determination of meaningful within-patient change for PRO scores based on anchor-based and distribution-based analyses

**Fig. 2:**
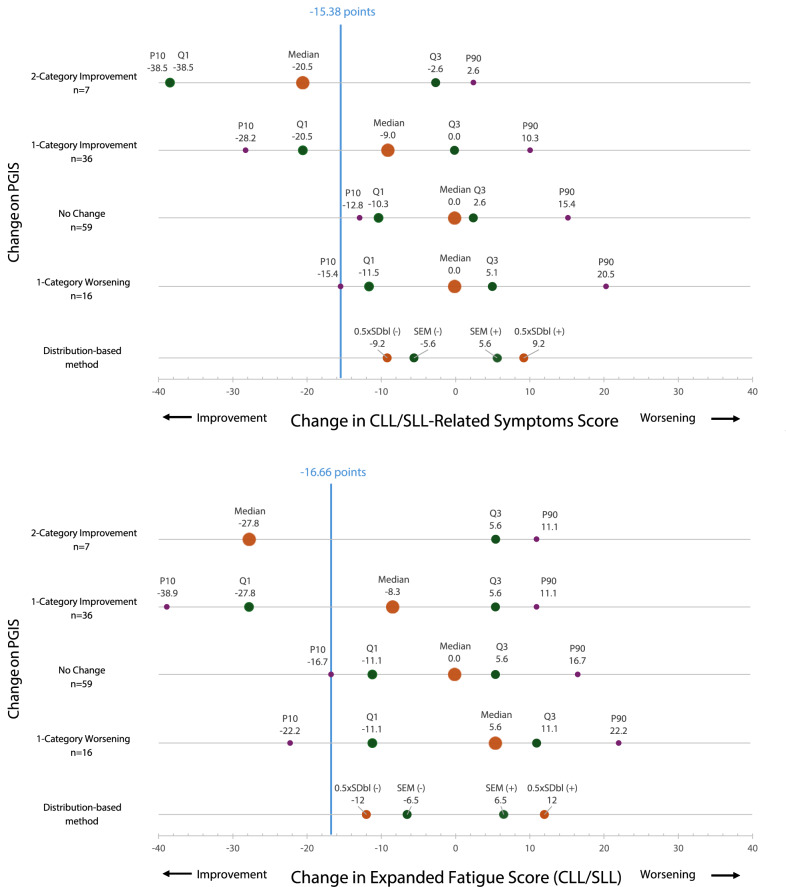
Summary of MWPC results for CLL/SLL-related Symptoms (upper panel) and Expanded Fatigue (lower panel) measures using anchor-based (PGI-S as an anchor) and distribution-based methods (CLL participants, 20020 ITT population). Key: for anchor categories, orange=median, green=1st quartile (Q1) and 3rd quartile (Q3), purple=10th percentile (P10) and 90th percentile (P90); for distribution-based method, orange=half standard deviation at baseline (0.5xSDbl), green=standard error of measurement (SEM)

**Fig. 3:**
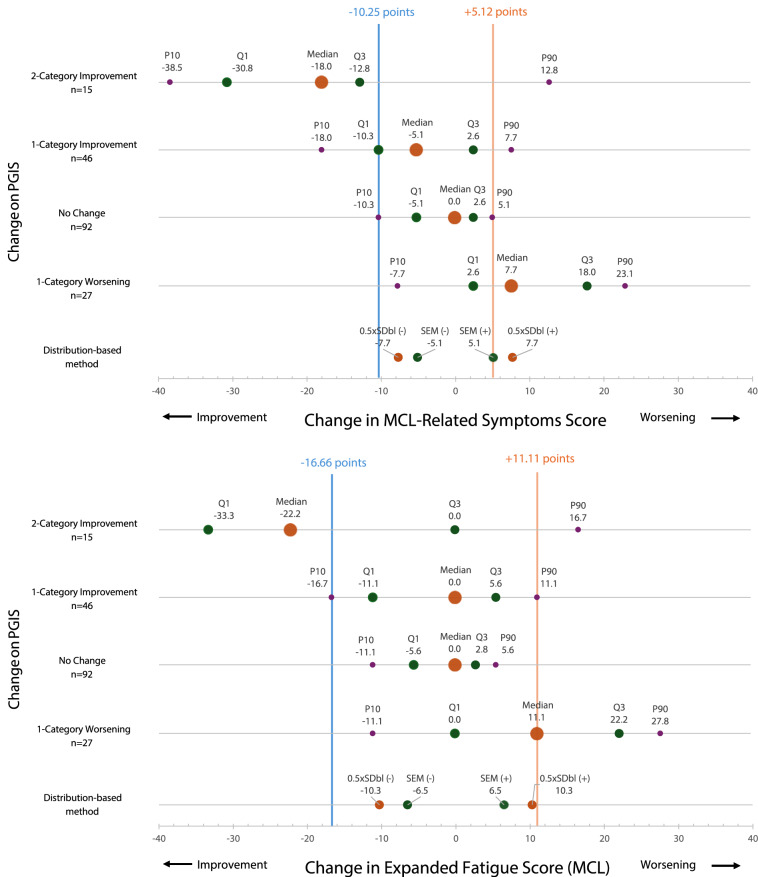
Summary of MWPC results for MCL-related Symptoms (upper panel) and Expanded Fatigue (lower panel) measures using anchor-based (PGI-S as an anchor) and distribution-based methods (MCL participants, 20019 ITT population). Key: for anchor categories, orange=median, green=1st quartile (Q1) and 3rd quartile (Q3), purple=10th percentile (P10) and 90th percentile (P90); for distribution-based method, orange=half standard deviation at baseline (0.5xSDbl), green=standard error of measurement (SEM)

The description of the change in scores of the three PRO measures across the categories of change in the PGI-S was explored to determine the amount of change that would best discriminate between groups of participants with no change (stable) versus those who improved or worsened according to the PGI-S. This was primarily achieved by examining the distributional statistics (median, quartiles, and 10th and 90th percentiles [P10 and P90]) of the change in scores between groups of participants with no change versus 1-category improvement and 1-category worsening based on the PGI-S. These summary statistics are displayed in Figs. 2 and 3 (full distributions are provided in the Suppl. Tables 9 and 10).

In participants with CLL, at least half of the participants with a 1-category improvement on the PGI-S had a decrease in CLL/SLL-related Symptoms score of 8.97 points or more (median), whereas just 10% of the participants with no change on the PGI-S had a decrease of 12.82 points or more (P10). For the Expanded Fatigue measure in participants with CLL, at least half of the participants with a 1-category improvement on the PGI-S had a decrease in score of 8.33 points or more (median), whereas only 25% of those with no change on the PGI-S reported a decrease of 11.11 points or more (first quartile: Q1). The distribution of scores in participants with CLL who experienced worsening based on the PGI-S was not clearly different from those with no change in PGI-S, so an estimate for meaningful within-patient worsening could not be supported with these data. Distribution-based estimates of meaningful change in this sample ranged between 5.6 (SEM [standard error of measurement]) and 9.2 (0.5SD [standard deviation]) for the CLL/SLL-related Symptoms score and 6.5 (SEM) and 12.0 (0.5SD) for the Expanded Fatigue score (thresholds estimated by distribution-based methods apply to both improvement and worsening).

In MCL participants, MCL-related Symptoms and Expanded Fatigue score distributions among those with a 1-category improvement on the PGI-S were largely overlapping with that observed in stable participants. At least half of the participants with a 2-category improvement in the PGI-S reported a decrease in the MCL-related Symptoms score of 17.95 points or more (median) and at least 75% reported a decrease of 12.82 points or more (third quartile: Q3). In contrast, just 10% of the participants with no change on the PGI-S reported a decrease in the MCL-related Symptoms score of at least 10.25 points (P10). At least half of the participants with a 1-category worsening in the PGI-S reported an increase in the MCL-related Symptoms score of 7.69 points or more (median), whereas in contrast, at least 25% of those who reported no change on the PGI-S reported an increase of 2.56 points or more (Q3). For the Expanded Fatigue score in participants with MCL, at least half of the participants with a 2-category improvement on the PGI-S reported a decrease in score of 22.22 or more (median), whereas just 10% of those with no change on the PGI-S reported a decrease of at least 11.11 points (P10). At least half of the participants with a 1-category worsening in the PGI-S reported an increase in the Expanded Fatigue score of 11.11 points or more (median), whereas in contrast, 10% of those who reported no change on the PGI-S reported an increase of 5.56 points or more (P90). Distribution-based estimates of meaningful change in this sample ranged between 5.1 (SEM) and 7.7 (0.5SD) for the MCL-related Symptoms score and 6.5 (SEM) and 10.3 (0.5SD) for the Expanded Fatigue score.

Triangulation of results from the anchor-based and distribution-based analysis in participants with CLL are summarized in Fig. 2 (CLL/SLL-related Symptoms, top, and Expanded Fatigue, bottom), and results are summarized for participants with MCL in Fig. 3 (MCL-related Symptoms, top, and Expanded Fatigue, bottom). Triangulation considered the uncertainty of the findings, variability of the score distributions within the PGI-S categories (to integrate individual-level uncertainty), and the range of obtained values for the threshold based on the different methods. This overall interpretation of the results led to the following recommendations for the MWPC estimates (rounded down at 2 decimal places in absolute value, to ensure observed value of change is included by applying definition): -15.38 and -10.25 for meaningful within-patient improvement in CLL/SLL-related Symptoms score and MCL-related Symptoms score respectively; +5.12 points for meaningful within-patient worsening in MCL-related Symptoms score while no estimate was available for the CLL/SLL-related Symptoms score; -16.66 for meaningful within-patient improvement in Expanded Fatigue score for both participants with CLL and those with MCL, and + 11.11 points for meaningful within-patient worsening in Expanded Fatigue among participants with MCL while it was not evaluable in participants with CLL.

## Discussion

The CLL/SLL-related Symptoms, MCL-related Symptoms, and Expanded Fatigue measures showed strong psychometric properties in the target clinical trial participants with CLL and MCL based on both CTT and RMT frameworks. These psychometric analysis results were mostly consistent with a previous analysis of a phase 1/2 trial data (LOXO-BTK-18001) in participants with CLL and MCL, which reinforces the strength of the present findings [30].

CTT analyses demonstrated adequate internal consistency and test-retest reliability in all three measures. The associations of the scores with measures of other associated concepts were as hypothesized, providing strong evidence of construct validity. All three measures demonstrated good ability to detect worsening, and to a lesser degree, improvement in symptoms as evaluated by the PGI-S anchor.

The results obtained in the RMT paradigm provided a finer understanding of the three measures, revealing some possible areas of attention when using them. The RMT results confirmed the adequacy of the response scale used in the three measures, as none showed disordered thresholds. Results also highlighted that the items composing the measures, especially Expanded Fatigue, were ordered in a meaningful way along the measured continuum. A possible weakness of the three measures identified in the RMT analyses was their suboptimal targeting to the sample of observations from the study, with a floor effect. No single item adequately captured the severity of symptoms experienced by participants with the lowest symptoms severity, which could hinder the detection of subtle improvements of mild symptoms. The floor effect may not, however, constitute a concern in a more advanced patient population where the objective is to detect improvement of more severe symptoms and/or where the endpoint is based on detecting worsening of symptoms. Additionally, in both symptom measures, items related to fatigue tended to be over-discriminating, suggesting that the symptom scores are driven by fatigue-related items. While fatigue symptoms were reported as the most bothersome based on qualitative research [4], it should be noted that symptom and fatigue scores are expected to be largely correlated, given the predominance of fatigue-related items in the symptom item set (6 out of 13). In the context of clinical trial results, this overlap should be emphasized to avoid overinterpretation, as findings from group comparisons (whether positive or negative) are likely to be aligned due to the shared item content between the scores.

Most of the items in the Expanded Fatigue measure showed good fit to the Rasch model, suggesting that they appropriately assess fatigue on a single continuum; however, the misfitting, non-fatigue-related items from the CLL/SLL- and MCL-related symptom measures do not support the existence of a single symptom continuum. Having under-discriminating fatigue-related items, combined with greater item-to-item and item-to-scale correlations with these items, potentially show that the disease-related symptom measures might be primarily driven by fatigue symptoms. These results may suggest that the CLL/SLL- and MCL-related Symptoms measures and sum scores of interest may function as indices (or composite scores) that assesses different concepts together. A set of six items measure fatigue symptoms while the other items measure various disease-related symptoms, which are not necessarily experienced together, depending on the overall level of symptoms severity. One caveat of a composite score is that there is no shared level of severity between participants; in other words, the severity of symptoms does not follow a single measurement continuum (e.g., some patients may experience more fatigue while others may experience more pain, but one does not necessarily follow the other). This heterogeneity could limit the interpretation of the composite score and comparability of symptom burden between participants (e.g., between treatment arms in a clinical trial). More broadly, the potential composite nature of these scores implies the need for a different perspective on the assessment of psychometric properties, including consideration of alternative measurement models for measure design, such as multiple cause indicator (formative) models [32]. One implication of this framework is the question of item weighting in score calculation, as equal weighting of all items—as applied in the present sum-score approach—may not always be appropriate. Assigning lower weights to fatigue-related items could mitigate their predominance in the assessment of overall symptoms (e.g., by taking accounting for correlations among fatigue-related items). While there is no standard approach for defining item weights, strategies based on the impact of individual items on external indicators (e.g., other COA scores reflecting impact on daily activities) could be further investigated [32]. Analyzing items as single-item outcomes may also represent a conceptually relevant alternative; however, this approach has well-recognized limitations, including poorer reliability and responsiveness.

Current analyses also supported recommended thresholds for MWPC for improvement and worsening. Estimates were defined considering the general uncertainty of the findings, which includes limited correlation between the scores and the anchor. Recommended thresholds, therefore, used a conservative approach for defining improvement and worsening in the context of expected treatment benefit (not overestimating improvement and minimizing worsening); being stringent enough to define improvement (setting a threshold sufficiently high), while doing the opposite for worsening (setting a threshold sufficiently low). For CLL/SLL-related Symptoms, a decrease of 15.38 points is recommended as a MWPC threshold for improvement while a threshold for worsening could not be determined as too few participants worsened according to the anchor used. For MCL-related Symptoms, a decrease of 10.25 points is recommended as a MWPC threshold for improvement, while increase of 5.12 points is recommended for meaningful within-patient worsening. For Expanded Fatigue, a decrease of 16.66 points is recommended as a MWPC threshold for improvement, while an increase of 11.11 points is recommended for meaningful within-patient worsening in both populations.

Several limitations should also be acknowledged. First, the limited number of participants who experienced worsening hindered the definition of a MWPC threshold for worsening based on the CLL/SLL-related Symptoms score. Future research could establish such a definition. In the absence of an empirically derived threshold, we recommend by default using a positive change of two “increments” as the MWPC (i.e., worsening of 1 category in at least 2 items, or of 2 categories in at least 1 item), based on published MWPC definitions for the QLQ-C30 [33]. This corresponds to a worsening of at least 5.12-point increase in CLL/SLL-related Symptoms score and results in a threshold similar to that established in the present analyses for MCL-related symptoms, whose item composition is mostly identical. Another potential option would be to use the first observable positive change in score value larger than 10 points, as is typically defined for scores derived from the EORTC QLQ C30 measure [34]. However, this approach would result in a change that is likely larger than MWPC (corresponding to an increase of 10.25 points for the CLL/SLL-related Symptoms score) and is therefore not recommended. Second, the floor effect that was observed in the present analysis might have impacted assessment of the psychometric properties and MWPC definitions for the three measures of interest. Lastly, as results from CTT analyses may be sample-dependent [35], replicating analyses in a more severe patient population with smaller-to-no floor effects could further result in better understanding of these psychometric properties.

## Conclusions and implications

Combined with previously published qualitative data, this research showed that CLL/SLL-related Symptoms, MCL-related Symptoms, and Expanded Fatigue measures are appropriate for use in clinical trials of participants with CLL and MCL. The symptoms measures, being potentially composite measures, should be used with careful consideration for the specific population and PRO endpoint measurement strategy of the trial.

## Supplementary Information

Below is the link to the electronic supplementary material.

Supplementary Material 1

## Data Availability

Eli Lilly and Company provides access to all individual participant data collected during the trial, after anonymization, except pharmacokinetic or genetic data. Data are available to request 6 months after the indication studied has been approved in the United States and European Union and after primary publication acceptance, whichever is later. No expiration date of data requests is currently set once data are made available. Access is provided after a proposal has been approved by an independent review committee identified for this purpose and after receipt of a signed data sharing agreement. Data and documents, including the study protocol, statistical analysis plan, clinical study report, blank or annotated case report forms, will be provided in a secure data sharing environment. For details on submitting a request, see the instructions provided at www.vivli.org.

## Abbreviations

BR: Bendamustine plus rituximab
cBTKi: Covalent Bruton tyrosine kinase inhibitor
CLL/SLL: Chronic lymphocytic leukemia/small lymphocytic lymphoma
CTT: Classical test theory
ECOG: Eastern Cooperative Oncology Group
EORTC: European Organization for Research and Treatment of Cancer
EOT: End of treatment
EQ-5D-5L: EuroQol 5 Dimension 5 Level
ES: Effect size
ICC: Intraclass correlation coefficient
IdelaR: Idelalisib plus eight infusions of rituximab
IL: Item Library
MCL: Mantle cell lymphoma
MWPC: Meaningful within-patient change
NHL: Non-Hodgkin lymphoma
P10: 10th percentile
P90: 90th percentile
PF: Physical functioning scale
PGI-C: Patient Global Impression of Change
PGI-S: Patient Global Impression of Severity
PRO: Patient-reported outcome
PSI: Person separation index
Q1: 1st quartile
Q3: 3rd quartile
QLQ-C30: Quality of Life Questionnaire Core 30
QLQ-CLL17: Quality of Life Questionnaires in patients with CLL
QLQ-NHL-HG29: Quality of Life Questionnaires in patients with non-Hodgkin lymphoma, high grade
QoL: Quality of life
RMT: Rasch measurement theory
SD: Standard deviation
SEM: Standard error of measurement
VAS: Visual analogue scale
