# Supplementary material for: Psychometric analysis of new lymphoma-specific patient-reported symptom measures derived from the EORTC item library
Source: J Patient Rep Outcomes. 2026 Jun 22;10:109. doi: 10.1186/s41687-026-01126-w (PMC13328598; doi:10.1186/s41687-026-01126-w)
Supplement: Supplementary file 1 — Supplementary Material 1 [file 41687_2026_1126_MOESM1_ESM.docx]

# Supplemental Materials

Table 4: Item-to-item and item-to-score correlations for CLL/SLL-related Symptoms at baseline

|  | | **Correlation coefficients between each item^[a]^** | | | | | | | | | | | | | **Item-to-Score Correlation** |
| --- | --- | --- | --- | --- | --- | --- | --- | --- | --- | --- | --- | --- | --- | --- | --- |
| **Item** | **N** | **QLQ8** | **QLQ9** | **QLQ10** | **QLQ11** | **QLQ12** | **QLQ13** | **QLQ14** | **QLQ18** | **IL1** | **IL2** | **IL3** | **IL4** | **IL5** |  |
| QLQ8: Short of Breath | 172 | 1.00 | . | . | . | . | . | . | . | . | . | . | . | . | 0.48 |
| QLQ9: Had Pain | 172 | 0.34 | 1.00 | . | . | . | . | . | . | . | . | . | . | . | 0.55 |
| QLQ10: Need to Rest | 172 | 0.48 | 0.57 | 1.00 | . | . | . | . | . | . | . | . | . | . | 0.77 |
| QLQ11: Trouble Sleeping | 172 | 0.29 | 0.47 | 0.59 | 1.00 | . | . | . | . | . | . | . | . | . | 0.54 |
| QLQ12: Felt Weak | 172 | 0.55 | 0.52 | 0.80 | 0.60 | 1.00 | . | . | . | . | . | . | . | . | 0.77 |
| QLQ13: Lacked Appetite | 172 | 0.32 | 0.33 | 0.56 | 0.33 | 0.60 | 1.00 | . | . | . | . | . | . | . | 0.49 |
| QLQ14: Felt Nauseated | 172 | 0.30 | 0.21 | 0.45 | 0.22 | 0.40 | 0.51 | 1.00 | . | . | . | . | . | . | 0.34 |
| QLQ18: Felt Tired | 172 | 0.63 | 0.45 | 0.82 | 0.55 | 0.82 | 0.57 | 0.48 | 1.00 | . | . | . | . | . | 0.80 |
| IL1: Felt Drowsy | 169 | 0.49 | 0.47 | 0.62 | 0.59 | 0.62 | 0.34 | 0.37 | 0.73 | 1.00 | . | . | . | . | 0.64 |
| IL2: Fevers or Chills | 169 | 0.39 | 0.32 | 0.38 | 0.25 | 0.53 | 0.40 | 0.36 | 0.47 | 0.40 | 1.00 | . | . | . | 0.36 |
| IL3: Lack of Energy | 169 | 0.63 | 0.59 | 0.79 | 0.61 | 0.83 | 0.61 | 0.54 | 0.88 | 0.71 | 0.58 | 1.00 | . | . | 0.83 |
| IL4: Night Sweats | 169 | 0.34 | 0.47 | 0.34 | 0.37 | 0.48 | 0.28 | 0.28 | 0.46 | 0.46 | 0.41 | 0.49 | 1.00 | . | 0.44 |
| IL5: Sudden Tiredness | 169 | 0.53 | 0.54 | 0.79 | 0.62 | 0.80 | 0.45 | 0.49 | 0.81 | 0.73 | 0.47 | 0.82 | 0.54 | 1.00 | 0.77 |
| [a] Polychoric correlation coefficient | | | | | | | | | | | | | | | |

Table 5: Item-to-item and item-to-score correlations for Expanded Fatigue (CLL participants) at baseline

|  | | **Correlation coefficients between each item^[a]^** | | | | | | **Item-to-Score Correlation** |
| --- | --- | --- | --- | --- | --- | --- | --- | --- |
| **Item** | **N** | **QLQ10** | **QLQ12** | **QLQ18** | **IL1** | **IL3** | **IL5** |  |
| QLQ10: Need to Rest | 172 | 1.00 | . | . | . | . | . | 0.78 |
| QLQ12: Felt Weak | 172 | 0.80 | 1.00 | . | . | . | . | 0.76 |
| QLQ18: Felt Tired | 172 | 0.82 | 0.82 | 1.00 | . | . | . | 0.81 |
| IL1: Felt Drowsy | 169 | 0.62 | 0.62 | 0.73 | 1.00 | . | . | 0.63 |
| IL3: Lack of Energy | 169 | 0.79 | 0.83 | 0.88 | 0.71 | 1.00 | . | 0.82 |
| IL5: Sudden Tiredness | 169 | 0.79 | 0.80 | 0.81 | 0.73 | 0.82 | 1.00 | 0.77 |
| [a] Polychoric correlation coefficient | | | | | | | | |

Table 6: Item-to-item and item-to-score correlations for MCL-related Symptoms at baseline

|  | | **Correlation coefficients between each item^[a]^** | | | | | | | | | | | | | **Item-to-Score Correlation** |
| --- | --- | --- | --- | --- | --- | --- | --- | --- | --- | --- | --- | --- | --- | --- | --- |
| **Item** | **N** | **QLQ8** | **QLQ9** | **QLQ10** | **QLQ12** | **QLQ13** | **QLQ14** | **QLQ18** | **IL1** | **IL2** | **IL3** | **IL4** | **IL5** | **IL6** |  |
| QLQ8: Short of Breath | 398 | 1.00 | . | . | . | . | . | . | . | . | . | . | . | . | 0.50 |
| QLQ9: Had Pain | 398 | 0.28 | 1.00 | . | . | . | . | . | . | . | . | . | . | . | 0.44 |
| QLQ10: Need to Rest | 398 | 0.55 | 0.51 | 1.00 | . | . | . | . | . | . | . | . | . | . | 0.71 |
| QLQ12: Felt Weak | 398 | 0.59 | 0.48 | 0.70 | 1.00 | . | . | . | . | . | . | . | . | . | 0.73 |
| QLQ13: Lacked Appetite | 398 | 0.53 | 0.30 | 0.51 | 0.66 | 1.00 | . | . | . | . | . | . | . | . | 0.53 |
| QLQ14: Felt Nauseated | 398 | 0.51 | 0.27 | 0.39 | 0.47 | 0.59 | 1.00 | . | . | . | . | . | . | . | 0.35 |
| QLQ18: Felt Tired | 398 | 0.64 | 0.49 | 0.86 | 0.81 | 0.59 | 0.54 | 1.00 | . | . | . | . | . | . | 0.79 |
| IL1: Night Sweats | 391 | 0.35 | 0.30 | 0.37 | 0.50 | 0.40 | 0.35 | 0.43 | 1.00 | . | . | . | . | . | 0.43 |
| IL2: Fevers or Chills | 391 | 0.44 | 0.25 | 0.35 | 0.39 | 0.45 | 0.46 | 0.41 | 0.51 | 1.00 | . | . | . | . | 0.33 |
| IL3: Lack of Energy | 391 | 0.55 | 0.42 | 0.74 | 0.80 | 0.63 | 0.47 | 0.86 | 0.49 | 0.47 | 1.00 | . | . | . | 0.77 |
| IL4: Felt Drowsy | 391 | 0.44 | 0.41 | 0.65 | 0.57 | 0.43 | 0.30 | 0.64 | 0.36 | 0.43 | 0.63 | 1.00 | . | . | 0.58 |
| IL5: Sudden Tiredness | 391 | 0.48 | 0.39 | 0.64 | 0.65 | 0.42 | 0.42 | 0.73 | 0.38 | 0.42 | 0.76 | 0.60 | 1.00 | . | 0.61 |
| IL6: Bloated Feeling in Abdomen | 391 | 0.41 | 0.49 | 0.51 | 0.44 | 0.42 | 0.37 | 0.54 | 0.44 | 0.28 | 0.54 | 0.49 | 0.44 | 1.00 | 0.48 |
| [a] Polychoric correlation coefficient | | | | | | | | | | | | | | | |

Table 7: Item-to-item and item-to-score correlations for Expanded Fatigue (MCL participants) at baseline

|  | | **Correlation coefficients between each item^[a]^** | | | | | | **Item-to-Score Correlation** |
| --- | --- | --- | --- | --- | --- | --- | --- | --- |
| **Item** | **N** | **QLQ10** | **QLQ12** | **QLQ18** | **IL3** | **IL4** | **IL5** |  |
| QLQ10: Need to Rest | 398 | 1.00 | . | . | . | . | . | 0.73 |
| QLQ12: Felt Weak | 398 | 0.70 | 1.00 | . | . | . | . | 0.71 |
| QLQ18: Felt Tired | 398 | 0.86 | 0.81 | 1.00 | . | . | . | 0.82 |
| IL3: Lack of Energy | 391 | 0.74 | 0.80 | 0.86 | 1.00 | . | . | 0.78 |
| IL4: Felt Drowsy | 391 | 0.65 | 0.57 | 0.64 | 0.63 | 1.00 | . | 0.58 |
| IL5: Sudden Tiredness | 391 | 0.64 | 0.65 | 0.73 | 0.76 | 0.60 | 1.00 | 0.63 |
| [a] Polychoric correlation coefficient | | | | | | | | |


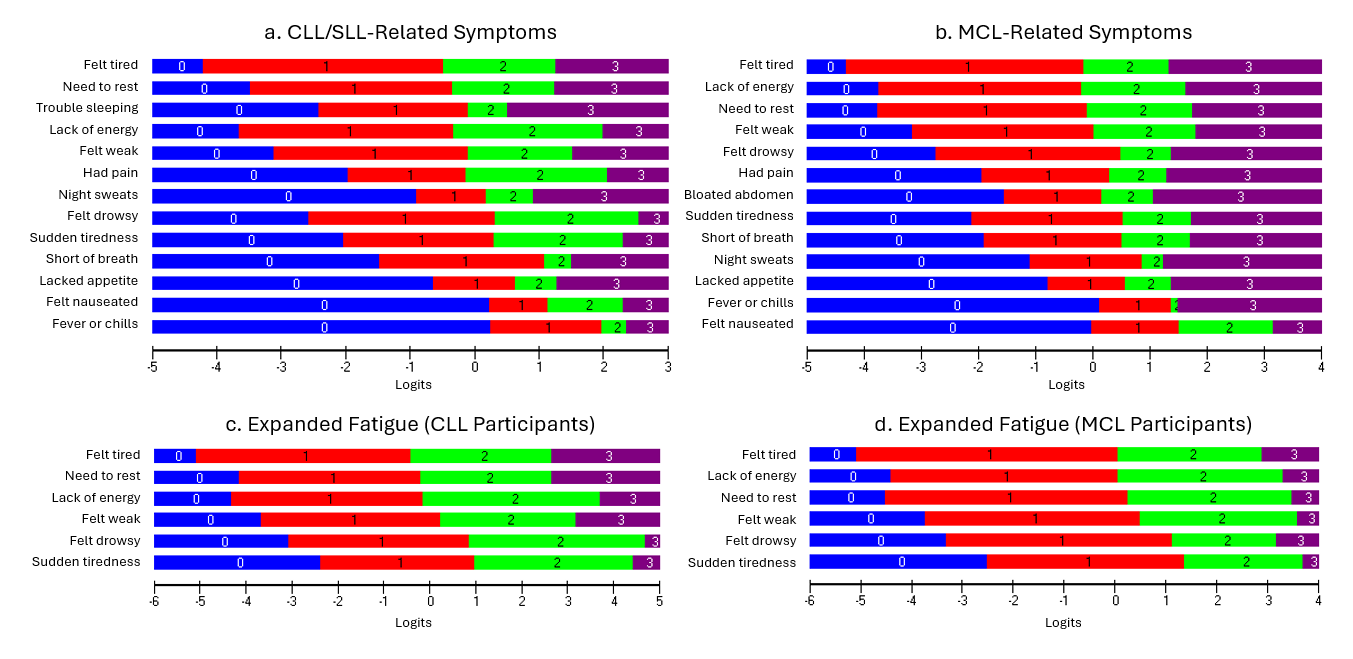
Figure 4: Item thresholds for CLL/SLL-related Symptoms (a), MCL-related Symptoms (b), and Expanded Fatigue for CLL participants (c) and for MCL participants (d)

Table 8: Item fit and fit residuals for CLL/SLL-related Symptoms

| **Item** | **Statement** | **Location** | **SE** | **Fit Residual** | **Chi²** | **Prob** |
| --- | --- | --- | --- | --- | --- | --- |
| QLQ8 | Short of Breath | 0.375 | 0.072 | 2.151 | 28.803 | 0.0007 |
| QLQ9 | Had Pain | -0.006 | 0.066 | 2.624 | 15.222 | 0.0850 |
| QLQ10 | Need to Rest | -0.862 | 0.066 | -5.355 | 24.297 | 0.0039 |
| QLQ11 | Trouble Sleeping | -0.672 | 0.061 | 6.518 | 88.148 | <.0001 |
| QLQ12 | Felt Weak | -0.562 | 0.067 | -5.876 | 34.598 | <.0001 |
| QLQ13 | Lacked Appetite | 0.424 | 0.071 | 0.277 | 13.831 | 0.1285 |
| QLQ14 | Felt Nauseated | 1.225 | 0.087 | 1.666 | 10.028 | 0.3482 |
| QLQ18 | Felt Tired | -1.141 | 0.069 | -6.66 | 46.401 | <.0001 |
| IL1 | Felt Drowsy | 0.095 | 0.071 | -0.279 | 11.578 | 0.2382 |
| IL2 | Fever or Chills | 1.522 | 0.097 | 1.358 | 17.384 | 0.0430 |
| IL3 | Lack of Energy | -0.658 | 0.07 | -6.679 | 43.704 | <.0001 |
| IL4 | Night Sweats | 0.065 | 0.066 | 4.038 | 44.804 | <.0001 |
| IL5 | Sudden Tiredness | 0.194 | 0.069 | -3.143 | 22.013 | 0.0088 |

NOTE: significant positive fit residuals indicate under-discrimination, and significant negative fit residuals indicate over-discrimination

Table 9: Item fit and fit residuals for MCL-related Symptoms

| **Item** | **Statement** | **Location** | **SE** | **Fit Residual** | **Chi²** | **Prob** |
| --- | --- | --- | --- | --- | --- | --- |
| QLQ8 | Short of Breath | 0.11 | 0.033 | 0.306 | 5.205 | 0.8161 |
| QLQ9 | Had Pain | -0.117 | 0.032 | 9.16 | 153.901 | <.0001 |
| QLQ10 | Need to Rest | -0.695 | 0.033 | -8.03 | 60.569 | <.0001 |
| QLQ12 | Felt Weak | -0.435 | 0.032 | -11.737 | 115.159 | <.0001 |
| QLQ13 | Lacked Appetite | 0.391 | 0.036 | 2.9 | 42.199 | <.0001 |
| QLQ14 | Felt Nauseated | 1.549 | 0.047 | 0.083 | 21.339 | 0.0112 |
| QLQ18 | Felt Tired | -1.037 | 0.033 | -16.369 | 299.415 | <.0001 |
| IL1 | Night Sweats | 0.339 | 0.036 | 9.947 | 292.443 | <.0001 |
| IL2 | Fevers or Chills | 1.001 | 0.045 | 5.074 | 75.703 | <.0001 |
| IL3 | Lack of Energy | -0.77 | 0.032 | -16.354 | 214.61 | <.0001 |
| IL4 | Felt Drowsy | -0.288 | 0.033 | 0.049 | 13.534 | 0.1399 |
| IL5 | Sudden Tiredness | 0.053 | 0.033 | -6.226 | 49.257 | <.0001 |
| IL6 | Bloated Abdomen | -0.102 | 0.032 | 10.437 | 273.723 | <.0001 |

NOTE: significant positive fit residuals indicate under-discrimination, and significant negative fit residuals indicate over-discrimination

Table 10: Item fit and fit residuals for Expanded Fatigue (CLL participants)

| **Item** | **Statement** | **Location** | **SE** | **Fit Residual** | **Chi²** | **Prob** |
| --- | --- | --- | --- | --- | --- | --- |
| QLQ10 | Need to Rest | -0.558 | 0.079 | -0.89 | 8.079 | 0.4258 |
| QLQ12 | Felt Weak | -0.084 | 0.08 | -0.652 | 6.704 | 0.5689 |
| QLQ18 | Felt Tired | -0.944 | 0.083 | -4.55 | 23.574 | 0.0027 |
| IL1 | Felt Drowsy | 0.825 | 0.083 | 4.127 | 17.025 | 0.0299 |
| IL3 | Lack of Energy | -0.246 | 0.083 | -4.5 | 15.396 | 0.0519 |
| IL5 | Sudden Tiredness | 1.008 | 0.082 | -0.605 | 26.142 | 0.0010 |

NOTE: significant positive fit residuals indicate under-discrimination, and significant negative fit residuals indicate over-discrimination

Table 11: Item fit and fit residuals for Expanded Fatigue score (MCL participants)

| **Item** | **Statement** | **Location** | **SE** | **Fit Residual** | **Chi²** | **Prob** |
| --- | --- | --- | --- | --- | --- | --- |
| QLQ10 | Need to Rest | -0.255 | 0.039 | -2.754 | 16.358 | 0.0220 |
| QLQ12 | Felt Weak | 0.121 | 0.037 | -4.296 | 13.031 | 0.0714 |
| QLQ18 | Felt Tired | -0.704 | 0.039 | -12.58 | 147.741 | <.0001 |
| IL3 | Lack of Energy | -0.349 | 0.038 | -10.904 | 106.268 | <.0001 |
| IL4 | Felt Drowsy | 0.335 | 0.038 | 9.68 | 141.658 | <.0001 |
| IL5 | Sudden Tiredness | 0.852 | 0.039 | 0.071 | 100.761 | <.0001 |

NOTE: significant positive fit residuals indicate under-discrimination, and significant negative fit residuals indicate over-discrimination

Table 12: Description of change in CLL/SLL-related Symptoms and Expanded Fatigue for CLL participants across categories of change in the PGI-S at Cycle 2

|  | **Change in PGI-S** | | | | | |
| --- | --- | --- | --- | --- | --- | --- |
| **Variable** | **≥3-category improvement N=4** | **2-category improvement N=7** | **1-category improvement N=36** | **No change N=59** | **1-category worsening N=16** | **2-category worsening N=1** |
| Change in CLL/SLL-related Symptoms score |  |  |  |  |  |  |
| n (missing) | 4 (0) | 7 (0) | 36 (0) | 59 (0) | 16 (0) | 1 (0) |
| Mean (SD) | -30.13 (23.07) | -20.15 (17.16) | -9.69 (14.26) | -1.29 (11.56) | 0.32 (13.79) | 20.51 (.) |
| Median | -29.49 | -20.51 | -8.97 | 0.00 | 0.00 | 20.51 |
| Q1, Q3 | -44.87, -15.38 | -38.46, -2.56 | -20.51, 0.00 | -10.26, 2.56 | -11.54, 5.13 | 20.51, 20.51 |
| P10, P90 | -58.97, -2.56 | -38.46, 2.56 | -28.21, 10.26 | -12.82, 15.38 | -15.38, 20.51 | 20.51, 20.51 |
| Min, Max | -58.97, -2.56 | -38.46, 2.56 | -38.46, 23.08 | -30.77, 33.33 | -17.95, 33.33 | 20.51, 20.51 |
|  |  |  |  |  |  |  |
| Change in Expanded Fatigue score (CLL/SLL participants) |  |  |  |  |  |  |
| n (missing) | 4 (0) | 7 (0) | 36 (0) | 59 (0) | 16 (0) | 1 (0) |
| Mean (SD) | -33.33 (27.59) | -21.43 (22.55) | -10.34 (20.67) | 0.09 (14.61) | 1.39 (16.67) | 22.22 (.) |
| Median | -33.33 | -27.78 | -8.33 | 0.00 | 5.56 | 22.22 |
| Q1, Q3 | -52.78, -13.89 | -44.44, 5.56 | -27.78, 5.56 | -11.11, 5.56 | -11.11, 11.11 | 22.22, 22.22 |
| P10, P90 | -66.67, 0.00 | -44.44, 11.11 | -38.89, 11.11 | -16.67, 16.67 | -22.22, 22.22 | 22.22, 22.22 |
| Min, Max | -66.67, 0.00 | -44.44, 11.11 | -55.56, 33.33 | -27.78, 50.00 | -27.78, 33.33 | 22.22, 22.22 |

Table 13: Description of change in MCL-related Symptom and Expanded Fatigue for MCL participants across categories of change in the PGI-S at week 9

|  | **Change in PGI-S** | | | | | | |
| --- | --- | --- | --- | --- | --- | --- | --- |
| **Variable** | **≥3-category improvement N=5** | **2-category improvement N=15** | **1-category improvement N=46** | **No change N=92** | **1-category worsening N=27** | **2-category worsening N=3** | **>=3-category worsening N=1** |
| Change in MCL-related Symptoms score |  |  |  |  |  |  |  |
| n (missing) | 5 (0) | 15 (0) | 46 (0) | 92 (0) | 27 (0) | 3 (0) | 1 (0) |
| Mean (SD) | -13.33 (19.05) | -19.83 (22.63) | -4.40 (10.36) | -1.76 (8.99) | 9.50 (11.26) | 0.85 (1.48) | 35.90 (.) |
| Median | -10.26 | -17.95 | -5.13 | 0.00 | 7.69 | 0.00 | 35.90 |
| Q1, Q3 | -17.95, 2.56 | -30.77,-12.82 | -10.26, 2.56 | -5.13, 2.56 | 2.56, 17.95 | 0.00, 2.56 | 35.90,35.90 |
| P10, P90 | -43.59, 2.56 | -38.46, 12.82 | -17.95, 7.69 | -10.26, 5.13 | -7.69, 23.08 | 0.00, 2.56 | 35.90,35.90 |
| Min, Max | -43.59, 2.56 | -76.92, 15.38 | -38.46,12.82 | -46.15,23.08 | -10.26, 41.03 | 0.00, 2.56 | 35.90,35.90 |
|  |  |  |  |  |  |  |  |
| Change in Expanded Fatigue score (MCL participants) |  |  |  |  |  |  |  |
| n (missing) | 5 (0) | 15 (0) | 46 (0) | 92 (0) | 27 (0) | 3 (0) | 1 (0) |
| Mean (SD) | -17.78 (28.71) | -21.48 (29.91) | -2.54 (13.34) | -1.39 (11.01) | 11.11 (15.10) | 3.70 (3.21) | 44.44 (.) |
| Median | 0.00 | -22.22 | 0.00 | 0.00 | 11.11 | 5.56 | 44.44 |
| Q1, Q3 | -33.33, 0.00 | -33.33, 0.00 | -11.11, 5.56 | -5.56, 2.78 | 0.00, 22.22 | 0.00, 5.56 | 44.44,44.44 |
| P10, P90 | -61.11, 5.56 | -44.44, 16.67 | -16.67, 11.11 | -11.11, 5.56 | -11.11, 27.78 | 0.00, 5.56 | 44.44,44.44 |
| Min, Max | -61.11, 5.56 | -100.00,27.78 | -38.89, 38.89 | -55.56, 33.33 | -27.78, 33.33 | 0.00, 5.56 | 44.44,44.44 |
